# Case Report: Whole-Exome Sequencing-Based Copy Number Variation Analysis Identified a Novel *DRC1* Homozygous Exon Deletion in a Patient With Primary Ciliary Dyskinesia

**DOI:** 10.3389/fgene.2022.940292

**Published:** 2022-07-06

**Authors:** Ying Liu, Cheng Lei, Rongchun Wang, Danhui Yang, Binyi Yang, Yingjie Xu, Chenyang Lu, Lin Wang, Shuizi Ding, Ting Guo, Shaokun Liu, Hong Luo

**Affiliations:** ^1^ Department of Pulmonary and Critical Care Medicine, The Second Xiangya Hospital, Central South University, Changsha, China; ^2^ Research Unit of Respiratory Disease, Central South University, Changsha, China; ^3^ Hunan Diagnosis and Treatment Center of Respiratory Disease, Changsha, China

**Keywords:** whole-exome sequencing, CNV, *DRC1*, primary ciliary dykinesia, multiple morphological abnormalities of the sperm flagella

## Abstract

**Objective:** Whole-exome sequencing (WES) based copy number variation (CNV) analysis has been reported to improve the diagnostic rate in rare genetic diseases. In this study, we aim to find the disease-associated variants in a highly suspected primary ciliary dyskinesia (PCD) patient without a genetic diagnosis by routine WES analysis.

**Methods:** We identified the CNVs using the “Exomedepth” package in an undiagnosed PCD patient with a negative result through routine WES analysis. RNA isolation, PCR amplification, and Sanger sequencing were used to confirm the variant. High-speed video microscopy analysis (HSVA) and immunofluorescence analysis were applied to detect the functional and structural deficiency of nasal cilia and sperm flagella. Papanicolaou staining was employed to characterize the morphology of sperm flagella.

**Results:** NC_000002.11(NM_145038.5): g.26635488_26641606del, c.156-1724_244-2550del, r.156_243del, p. (Glu53Asnfs*13), a novel *DRC1* homozygous CNV, was identified by WES-based CNV analysis rather than routine variants calling, in a patient from a non-consanguineous family. HSVA results showed no significant change in ciliary beating frequency but with reduced beating amplitude compared with normal control, and his spermatozoa were almost immotile. The diagnosis of multiple morphological abnormalities of the sperm flagella (MMAF) was established through sperm motility and morphology analysis. PCR amplification and Sanger sequencing confirmed the novel variant of *DRC1*. Immunofluorescence showed that both cilia and sperm flagella were deficient in protein expression related to the dynein regulatory complex.

**Conclusion:** This report identifies a novel *DRC1* disease-associated variant by WES-based CNV analysis from a highly suspected PCD patient with MMAF. Our findings not only expand the genetic spectrum of PCD with MMAF but suggest that in combination with CNV analysis might improve the efficiency of genetic tests.

## Introduction

Primary ciliary dyskinesia (PCD, MIM 244400) is a disease mainly inherited in an autosomal recessive manner and primarily caused by variants in genes required for transport, assembly, and function in motile cilia (Lucas et al., 2020). Due to motile cilia distributed mainly in the respiratory and reproductive system, most PCD patients exhibited bronchiectasis, chronic sinusitis, and infertility (Bhatt and Hogg, 2020). With increasing knowledge of the genetic background in PCD, the prevalence of PCD has been estimated up to at least 1:7,500 (Hannah, 2022). According to the guidelines published by the European Respiratory Society (ERS), the diagnosis of PCD requires the use of multiple methods (Lucas et al., 2017). These include the measurement of nasal nitric oxide (nNO), direct analysis of ciliary beat frequency and pattern by high-speed video microscopy analysis (HSVA), followed by the confirmatory method, including transmission electron microscope (TEM) analysis for analyzing the characteristic defects in ciliary ultrastructure, and genetic analysis for identifying biallelic pathogenic variants. However, TEM has some limitations that cannot be ignored when used as a diagnostic tool (Werner and Kouis, 2017). Only three kinds of ciliary ultrastructure defects were regarded to be the diagnostic characteristics for PCD, and the diagnostic rate of TEM is relatively low (Shoemark, 2017). Therefore, genetic analysis plays an indispensable role in PCD diagnosis.

With the continual developments in sequencing techniques and bioinformatic analysis, whole-exome sequencing (WES) provides a powerful tool to confirm the diagnosis of PCD. WES technology can help detect deleterious genetic variants in nearly the entire coding region of the genome. So far, over 50 genes have been reported to cause PCD (Wallmeier et al., 2020), yet the genetic basis of the disease remains unknown in about 30% of suspected PCD patients (Bustamante-Marin et al., 2020). Routine WES analysis in PCD most often focuses on identifying single-nucleotide variations (SNVs) and short insertions and deletions (INDELs), but as another kind of human genetic variation, copy number variations (CNVs), which also play an indispensable role in human Mendelian rare genetic disease (Pös et al., 2021). Meanwhile, the relationship between PCD and CNVs has not been clearly studied.

In this study, we found a patient who presented with highly suspected PCD symptoms but without PCD-associated biallelic pathogenic variants identified by WES-based SNV and INDEL analysis. Then we conducted WES-based CNV analysis and identified a novel dynein regulatory complex subunit 1 (*DRC1*) homozygous CNV. As a central component of the nexin-dynein regulatory complex (N-DRC), *DRC1* can conjugate peripheral A and adjacent B microtubule to sustain regular ciliary motility (Wirschell et al., 2013). *DRC1* variants, detected using routine WES analysis, resulting in DRC1 protein loss of function and consequently PCD with multiple morphological abnormalities of the sperm flagella (MMAF), have been reported in recent studies (Lei et al., 2022). In our study, we identified a novel *DRC1* CNV that can also cause PCD and MMAF, but the PCD-associated biallelic pathogenic variant could not be detected by routine WES analysis initially. Our study showed that the WES-based CNV detecting approach may be an assistant way to improve PCD genetic accuracy.

## Materials and Methods

### Ethical Compliance

The review board of the second Xiangya Hospital of Central South University approved this study. Written informed consent was obtained from the patient and the healthy control.

### Routine Whole-Exome Sequencing Analysis

EDTA anti-coagulated venous blood was collected from the patient, the patient’s parents, and a healthy control. The genomic DNA was extracted using the QIAamp DNA Blood Mini Kit following the manufacturer’s protocol. Whole exome enrichment was performed using xGen Exome Research Panel v2 and sequenced with the Illumina NovaSeq® systems.

The sequenced reads were aligned to the reference genome (hg19) using BWA MEN, and PCR duplicates were marked with PICARD. Variants were called by HaplotypeCaller in GATK4.0 with default parameters, and retained considering DP (reads depth) ≥ 10, MQ (Mapping Quality) ≥ 30, and GQ (Genotyping Quality) ≥ 20. After annotation using ANNOVAR, variants both in coding region and splicing site were kept and synonymous variants were removed. Beyond that, variants were filtered by allele frequencies (AF) in the 1000 genome project, the Genome Aggregation Database (gnomAD v2.1.1), the NHLBI GO Exome Sequencing Project (ESP) and a local AF database with threshold of 1%, and potential damaging effect (predicted to be deleterious by at least 2 predictive tools among SIFT, Polyphen2-HVAR, MutationTaster and CADD for variants in coding region; dbscSNV score > 0.6 for variants in splicing site.

### Copy Number Variation Analysis

CNVs were called from the read depth of WES data using the ExomeDepth package according to the developers’ guidelines. ExomeDepth is a validated method for exome read-depth analysis, generating normalized read counts of the test sample by using an optimized set of reference samples as a comparison to determine the presence of a CNV at the exon level (Royer-Bertrand et al., 2021). Each exome was compared with a set of matched, aggregate reference samples for these analyses.

### RNA Isolation, PCR and Sanger Sequencing

We collected nasal epithelial biopsy sample from the patient and a healthy control and extracted total RNA using a GeneJET RNA Purification Kit (K0731, Thermo Fisher Scientific, Waltham, MA, United States) according to the manufacturer’s instructions. Then, we used TranScript® One-step gDNA Removal and cDNA synthesis SuperMix (AT311, Transgene) to synthesize cDNA. To confirm the deletion identified by CNV analysis, cDNA of *DRC1* were amplified using the GoldenStar® T6 Polymerase (TSE101, Tsingke). Primers were designed using the primer blast of NCBI (https://www.ncbi.nlm.nih.gov/tools/primer-blast/). The sequences of the primers are listed as following: forward primer: 5′-GAG​CAC​TTG​TCC​ACC​CAG​ATT-3′, reverse primer: 5′-GTA​TTG​AGC​ATT​TCC​CAC​AGC-3′. Meanwhile, according to the manufacturer’s instructions, genome DNA was obtained using QIAamp DNA Blood Mini Kit from the patient, patient’s parents, and healthy control. PCR and Sanger sequencing were performed to validate the *DRC1* breakpoint. The primer sequences designed are listed as follows: forward primer: 5′- GAG​CAG​GGT​CTT​GAT​GAT​GTA​A-3′, reverse primer: 5′- CAC​CTT​TAT​GAG​ATC​CAG​GGA​AA-3′.

### High-Speed Video Microscopy Analysis

Nasal brush biopsy samples were imaged using an upright Olympus BX53 microscope (Olympus, Tokyo, Japan) and recorded using a scientific complementary metal oxide semiconductor camera (Prime BSI, Teledyne Photometrics Inc., United States) as previously described (Xu et al., 2022).

### Sperm Morphological Analysis

Semen samples were collected from the patient after at least five days of sexual abstinence. According to the World Health Organization guideline to classify sperm flagella morphology (Cooper et al., 2010). Abnormal flagella of the sperm were classified as absent, short, bent, coiled, or irregular using Papanicolaou staining (Auger et al., 2016). Based on the morphology of the sperm flagella, each spermatozoon can only be classified in one morphological category.

### Immunofluorescence

Nasal epithelial tissues and sperm were fixed in 4% paraformaldehyde. Immunofluorescence on the slides was performed as described previously (Xu et al., 2022). Briefly, the slides were incubated overnight at 4°C with the primary antibodies DRC4 (HPA041311, 1:50, Sigma-Aldrich, Missouri, United States), and anti-acetylated tubulin (T7451, 1:500, Sigma-Aldrich, Missouri, United States). Then secondary antibodies detected the antibody binding, including Alexa Fluor 488 anti-mouse IgG (A-21121, 1:200, Invitrogen, Carlsbad, CA, United States) and Alexa Fluor 555 anti-rabbit IgG (A31572, 1:400, Invitrogen, Carlsbad, CA, United States). After incubation for 2 h at 37°C, all the slides were stained with 2-(4-aminophenyl)-1H-indole-6-carboxamidine (DAPI) for 5 min at room temperature. Fluorescence signals were recorded using an Olympus BX53 microscope (Olympus, Tokyo, Japan) and scientific complementary metal oxide semiconductor (sCMOS) camera (Prime BSI, Teledyne Photometrics Inc., United States).

## Results

### Case Presentation

**FIGURE 1:**
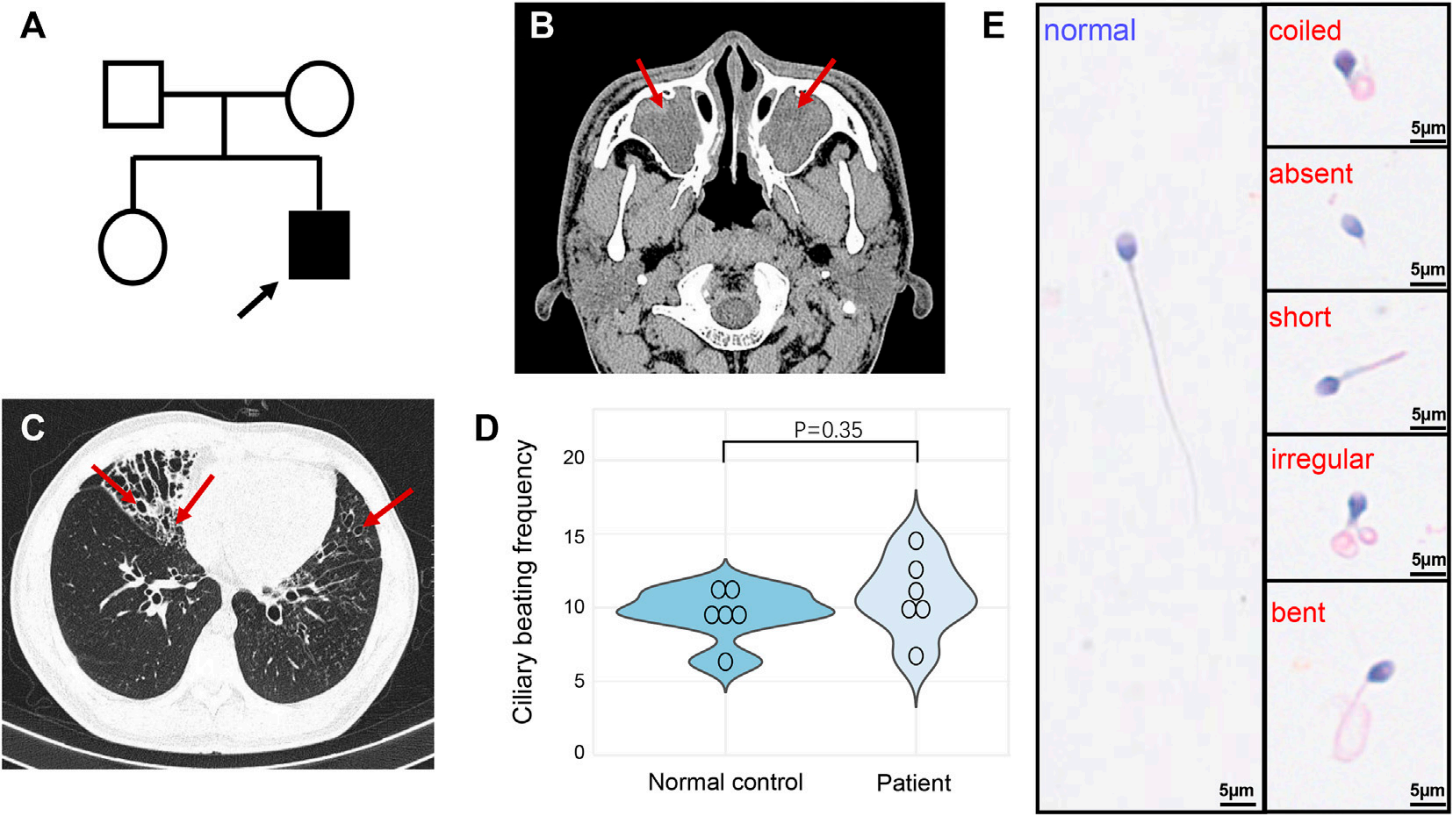
Pedigree and clinical features of the patient. **(A)** The pedigree indicated that the patient was from a non-consanguineous family, and other family members were asymptomatic. The arrow indicates the proband. **(B)** High resolution computed tomography of sinuses exhibited sinusitis (arrows). **(C)** Chest high-resolution computed tomography showed bronchiectasis in both lungs (arrows). **(D)** The ciliary beating frequencies showed no statistically significant difference between normal control and patient. **(E)** Papanicolaou staining revealed the abnormal morphology of sperm flagella compared with the healthy control.

The proband is a 19-year-old unmarried Chinese male with non-consanguineous parents and a healthy sister (Figure 1A). He was reported to have coughing, yellowish sputum, and sinusitis when he was three years old, and since then, the symptoms have been recurrent, and combined with exertional dyspnea. High resolution computed tomography revealed chronic sinusitis (Figure 1B), and bronchiectasis of both lungs (Figure 1C). The lung function test showed mild obstructive ventilatory impairment (predicted forced expiratory volume during the first second (FEV1): 79.3%, FEV1/forced vital capacity: 87.0%). In addition, nNO examination exhibited an abnormal low concentration (10.8 nL/min). HSVA of nasal brush biopsies showed that a normal ciliary beating frequency and a reduced amplitude in ciliary beating pattern (Figure 1D and Supplementary Video S1) compared with normal control (Supplementary Video S2). We also tested the motile function of the patient’s sperm, which showed that the patient’s spermatozoa were totally immotile (Table 1). Subsequent Papanicolaou staining also confirmed that the flagella morphologies of the patient were abnormal, which met the diagnostic criteria of MMAF (Table 1; Figure 1E).

**TABLE 1:** Semen parameters and sperm flagella morphology in the patient.

	Patient	References value
Semen parameters		
Semen volume (ml)	3.6	>1.5
Sperm count (10^6^/ml)	>2	>15.0
Motility (%)	0	>40.0
Progressive motility (%)	0	>32.0
Sperm morphology		
Normal flagella (%)	3.4	>23.0
Coiled (%)	64.9	<17.0
Short (%)	7.7	<1.0
Absent (%)	7.2	<5.0
Bent (%)	10.6	<13.0
Irregular (%)	6.2	<2.0

At least 200 spermatozoa were observed for morphology analysis.

### Whole-Exome Sequencing-Based Copy Number Variation Analysis to Capture Disease-Causing Gene Variations

**FIGURE 2:**
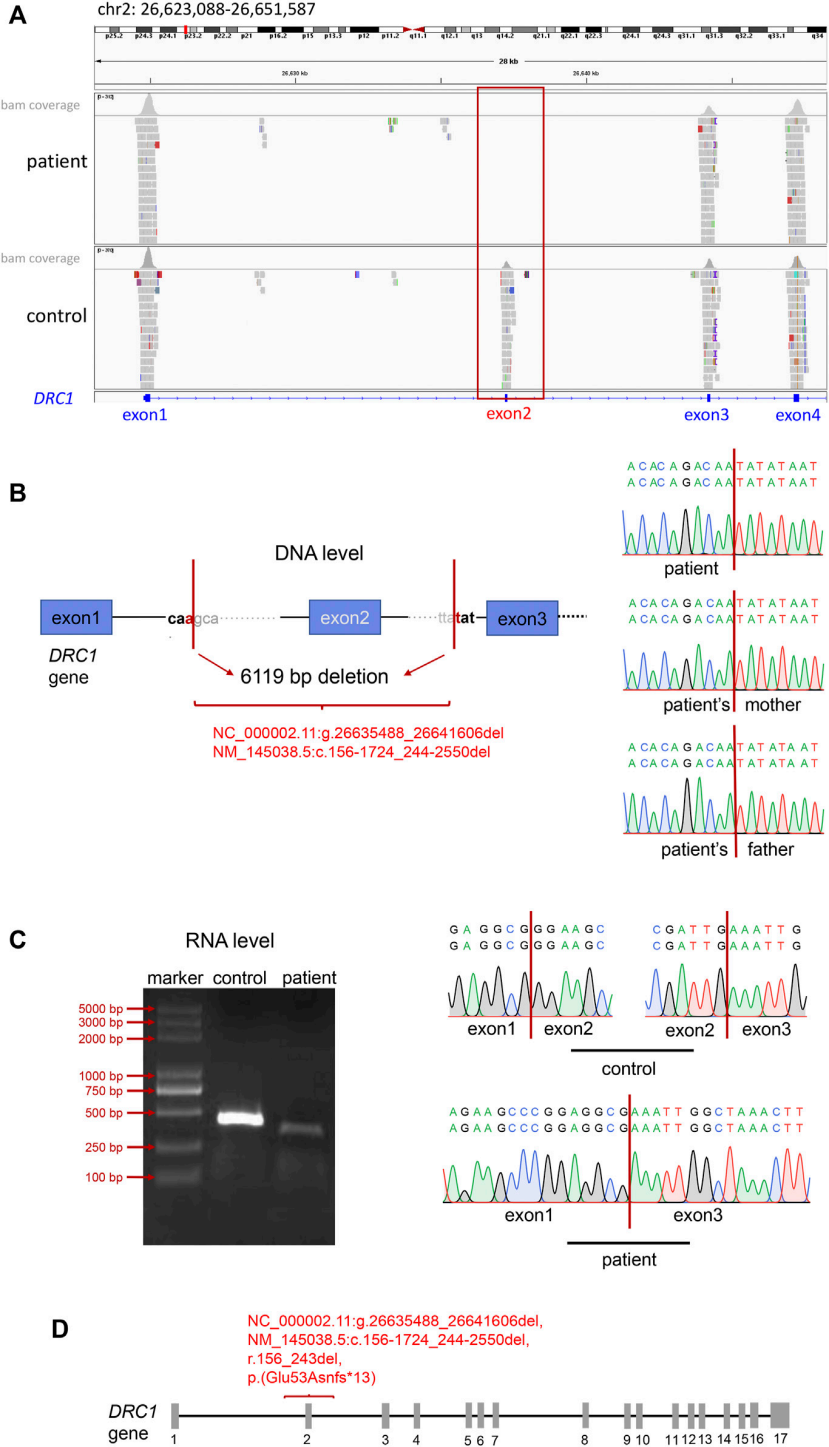
Identification of the *DRC1* variant. **(A)** IGV tools revealed the absence of exon 2 in *DRC1*. **(B)** Sanger sequencing showed the 6119 bp deletion of *DRC1* in the patient and the patient’s mother at DNA level. **(C)** Electrophoresis and Sanger sequencing identified the deletion of *DRC1* exon 2 in the patient compared with normal at RNA level. **(D)** The location of the novel *DRC1* homozygous variant in this report.

Combined with the above clinical symptoms of the patient: recurrent cough and sputum expectoration, nasal congestion since early childhood, abnormally low nNO levels, reduced ciliary beating amplitude, and MMAF phenotype, we highly suspected that he had PCD. Later, we performed WES-based SNV and INDEL analysis following PCD diagnostic criteria, and the filtering process was shown in Supplementary Figure S1. However, we did not identify any PCD-associated biallelic pathogenic variants (Supplementary Table S1). CNV analysis based on WES has been established to broaden the diagnostic rate in genetic disorders, so we further conducted CNV analysis. The results returned a homozygous deletion in exon 2 of *DRC1* in this patient (Figure 2A). Further PCR amplification and Sanger sequencing validated the homozygous absence of *DRC1* exon 2 at RNA level (Figure 2C). We next verified the breakpoint of the *DRC1* variant at the DNA level. Sanger sequencing of PCR product showed homozygous deletion of 6119 bp and the breakpoints were: NC_000002.11(NM_145038.5): g.26635488_26641606del, c.156-1724_244-2550del, r.156_243del, p. (Glu53Asnfs*13) in this patient (Figure 2B and Supplementary Figure S2). Yet, the results revealed the patient’s parents were all heterozygous carriers (Supplementary Figure S2).

### Analysis of Respiratory Cilia and Sperm Flagella

**FIGURE 3:**
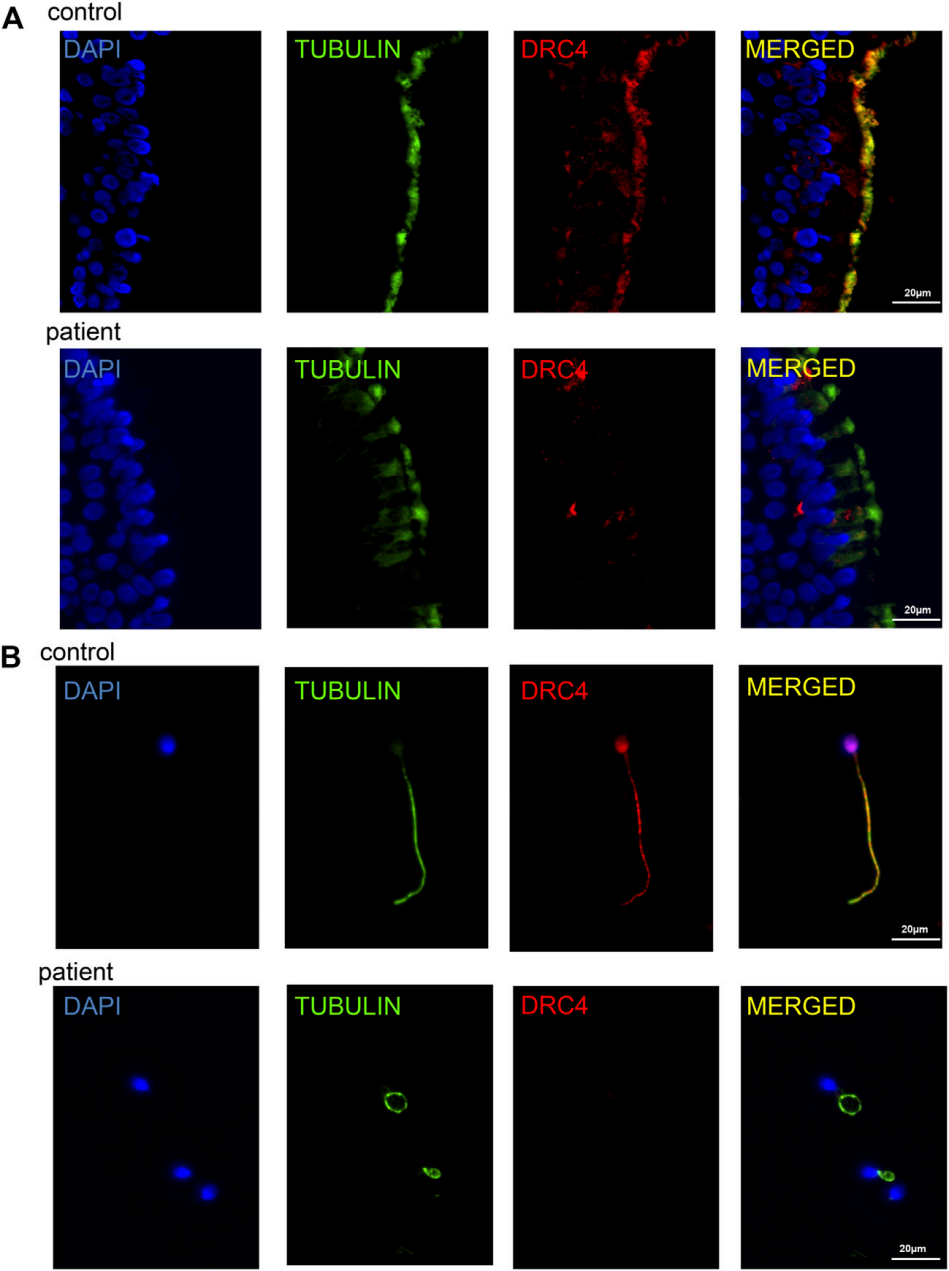
Analysis of ciliary tissue and sperm flagellum. **(A)** immunofluorescence analysis showed the absence of DRC4 protein expression in ciliated tissue of the patient compared with normal control. **(B)** immunofluorescence analysis showed the absence of DRC4 protein expression in sperm flagella of the patient compared to normal control.

Previously, it has been reported that the DRC3 or DRC4 protein deletion can confirm the deletion of DRC1 protein through immunofluorescence analysis (Wirschell et al., 2013). Since we do not have a suitable DRC1 antibody, we used the DRC4 antibody (also named *GAS8*, *GAS11*) for immunofluorescence analysis. The results confirmed the absence of DRC4 protein expression in the ciliated tissue of the patient compared with the healthy control (Figure 3A); immunofluorescence of the sperm flagellum also confirmed the deficiency of DRC4 expression in the patient (Figure 3B). All these results suggested that the patient had DRC1 deficiency.

## Discussion

Our study recruited a patient with bronchiectasis, chronic sinusitis, abnormal sperm motility, and abnormal sperm flagellar morphology. The HSVA, nNO, and semen analysis results suggested that the patient has highly clinical suspicion of PCD combined with MMAF. We initially conducted the routine WES analysis to identify the disease-causing variants in this patient, but we got a negative result. Then we performed WES-based CNV analysis, which is less noticed in variants calling and filtering procedures. Finally, a novel homozygous *DRC1* CNV was identified. Subsequent PCR amplification, Sanger sequencing, and immunofluorescence supported the pathogenicity of the CNV in *DRC1*. Although the transmission electron microscopic analysis results were not available, the diagnosis of PCD was made because we identified biallelic pathogenic *DRC1* variants in this patient.

According to PCD diagnostic guidelines, since not all patients have the characteristic abnormal ciliary axonemal ultrastructure and beating pattern in HSVA, it is crucial to identify disease-associated biallelic variants for patients with clinically suspected PCD (Lucas et al., 2017). Currently, among the high-throughput sequencing methods to identify PCD pathogenic variants, the WES analysis is the first-line method in China (Zhao et al., 2021). Though WES analysis covers 1–1.5% of the human genome, it houses approximately all exons of the known protein-coding genes (Ng et al., 2009). Compared with other next-generation sequencing technologies, such as whole-genome sequencing (WGS), WES is a more affordable high-throughput technology that allows the analysis of the coding regions of more than 20,000 genes (Tetreault et al., 2015). Routine WES analysis mainly considered SNVs and INDELs, but the prevalence and clinical significance of CNVs in PCD genes are yet unclear. Until now, only two studies have been conducted to study the relationship between CNVs and PCD. Marshall reported that by combining WES and the targeted CNV method, the genetic diagnosis rate of PCD could increase from 42% to 76% (Marshall et al., 2015). A study from Japan found that CNVs might play an important role in PCD, and CNVs in *DRC1* were the main cause of PCD in the Japanese population (Takeuchi et al., 2020). All the above research indicated that CNVs were significant as PCD disease-causing variants.

Human genetic disorders may arise from genetic variations ranging from the whole chromosome down to SNV. For humans, compared with SNVs and INDELs (smaller than 50 bp), CNVs account for only a tiny fraction (Lappalainen et al., 2019). Traditional CNV assessment mainly contains genome-wide screening technologies such as comparative genomic hybridization-microarray (arrayCGH) or locus CNV detection based on targeted PCR, for instance, multiplex ligation-dependent probe amplification (MLPA) (Zech et al., 2021). However, these two methods are time-consuming and relatively expensive, and each one cannot fully cover all CNV fragments (Falzarano et al., 2015; Roca et al., 2019). In the last decade, WES-based CNV analysis has been used for detecting CNVs, and it can overcome some of these shortcomings. Firstly, it permits concurrently detection of large and small CNVs (as previously detected by array CGH and MLPA, respectively) (Roca et al., 2019). Secondly, it is practical as it allows to determine SNVs, INDELs, and CNVs simultaneously, thus eliminating the necessity of using multiple different techniques in one patient and helping speed up the diagnostic process (Royer-Bertrand et al., 2021). Among WES-based CNV approaches, the “Exomedepth” package has been corroborated to have higher sensitivity and efficiency in detecting rare CNVs (Roca et al., 2019; Rajagopalan et al., 2020), and it is most used to identify CNVs in neurological diseases and mental disorders (Szatkiewicz et al., 2020; Cheng et al., 2021; Yu et al., 2021). Our study also used the “Exomedepth” package to call CNVs in a patient with a negative routine WES analysis result. The patient was finally confirmed to have a homozygous deletion of 6119 bp in *DRC1*, (NM_145038.5:c.156-1724_244-2550del), which contains exon 2 absence. The results suggested that WES-based CNV may help improve the diagnostic yield in highly suspected PCD.

The N-DRC functions as a linker between neighboring doublet microtubules, stabilizes the axonemal core structure, and serves as a central hub for controlling cilia motility (Gui et al., 2019). In most species, the N-DRC contains at least eleven, mostly well evolutionarily conserved subunits, *DRC1–11* (Osinka et al., 2019). Available data suggested that the 3-subunit core-complex (*DRC1/2/4*) of the N-DRC subunits is a scaffold for the assembly of functional subunits (*DRC3/5* and *DRC8/11*) (Gui et al., 2019). By now, DRC subunits deficiencies have been proved to cause motile ciliopathies. *DRC1*, *DRC2*, and *DRC4* were confirmed to be associated with PCD (Horani et al., 2013; Wirschell et al., 2013; Olbrich et al., 2015), and *DRC5* variants have been linked to asthenospermia and male infertility (Zhou, 2022). Variants in *DRC1* or *DRC2* could cause PCD and result in the loss of DRC4 protein expression, while *DRC4* does not affect the protein expression of DRC1 and DRC2 (Horani et al., 2013; Wirschell et al., 2013; Olbrich et al., 2015). *DRC1*, also known as *CCDC164*, locates on chromosome 2, consisting of 17 exons with 740 amino acids (Lei et al., 2022). A recent study found that *DRC1* variants could lead to PCD and MMAF, which is the same diagnosis as the patient we reported in this study (Lei et al., 2022). So far, only several genes have been covered to be related to PCD with MMAF, including *SPEF2*, *CFAP74*, *BRWD1*, *CCDC39*, *CCDC40*, *ARMC4*, and *DRC1* (Sha et al., 2020; Tu et al., 2020; Chen et al., 2021; Gao et al., 2021; Guo et al., 2021; Lei et al., 2022; Xu et al., 2022). Since the axonemal ultrastructure of respiratory cilia and sperm flagella is highly consistent, it is necessary to consider their sperm motility and morphology when diagnosing a patient with PCD. These findings provide strong evidence to confirm that the *DRC1* novel CNV is associated with PCD and MMAF.

In conclusion, we identified a novel homozygous variant of *DRC1* in a patient with PCD and MMAF by WES-based CNV analysis, while heterozygous in the patient’s parents. Moreover, the novel *DRC1* variant (NM_145038.5:c.156-1724_244-2550del), can be found in Esat Asian populations of gnomAD SVs v2.1 database once (https://gnomad.broadinstitute.org/variant/DEL_2_15666?dataset=gnomad_sv_r2_1). Previous studies have always potted the importance of detecting SNVs and INDELs based on WES, often ignoring CNVs. Our study shows that WES-based CNV analysis is a helpful adjunct method for identifying disease-associated variants in highly suspected PCD patients and increases the appropriateness of WES as a first-line genetic diagnostic method for PCD. Further studies could be conducted on the importance of CNVs in PCD, and our results suggest that if routine WES testing cannot detect the PCD-associated pathogenic variants, WES-based CNV analysis can be considered, thus perhaps further improving the PCD diagnostic rate.

## Data Availability

The datasets for this article are not publicly available due to concerns regarding participant/patient anonymity. Requests to access the datasets should be directed to the corresponding authors.
