# Supplementary material for: Case Report: Whole-Exome Sequencing-Based Copy Number Variation Analysis Identified a Novel DRC1 Homozygous Exon Deletion in a Patient With Primary Ciliary Dyskinesia
Source: Front Genet. 2022 Jul 6;13:940292. doi: 10.3389/fgene.2022.940292 (PMC9298917; doi:10.3389/fgene.2022.940292)
Supplement: Supplementary file 4 [file Image1.pdf]

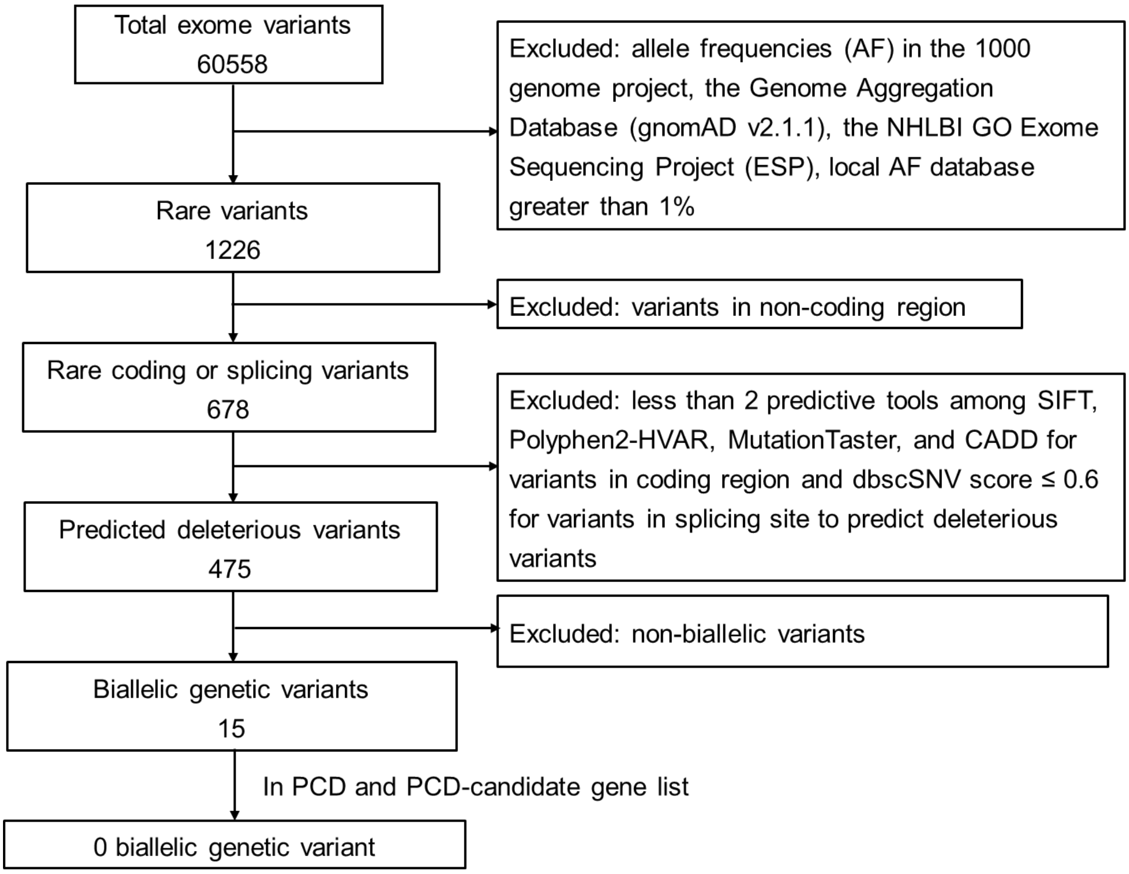

Supplementary figure 1. The variant filtering strategy for whole-exome sequencing data.

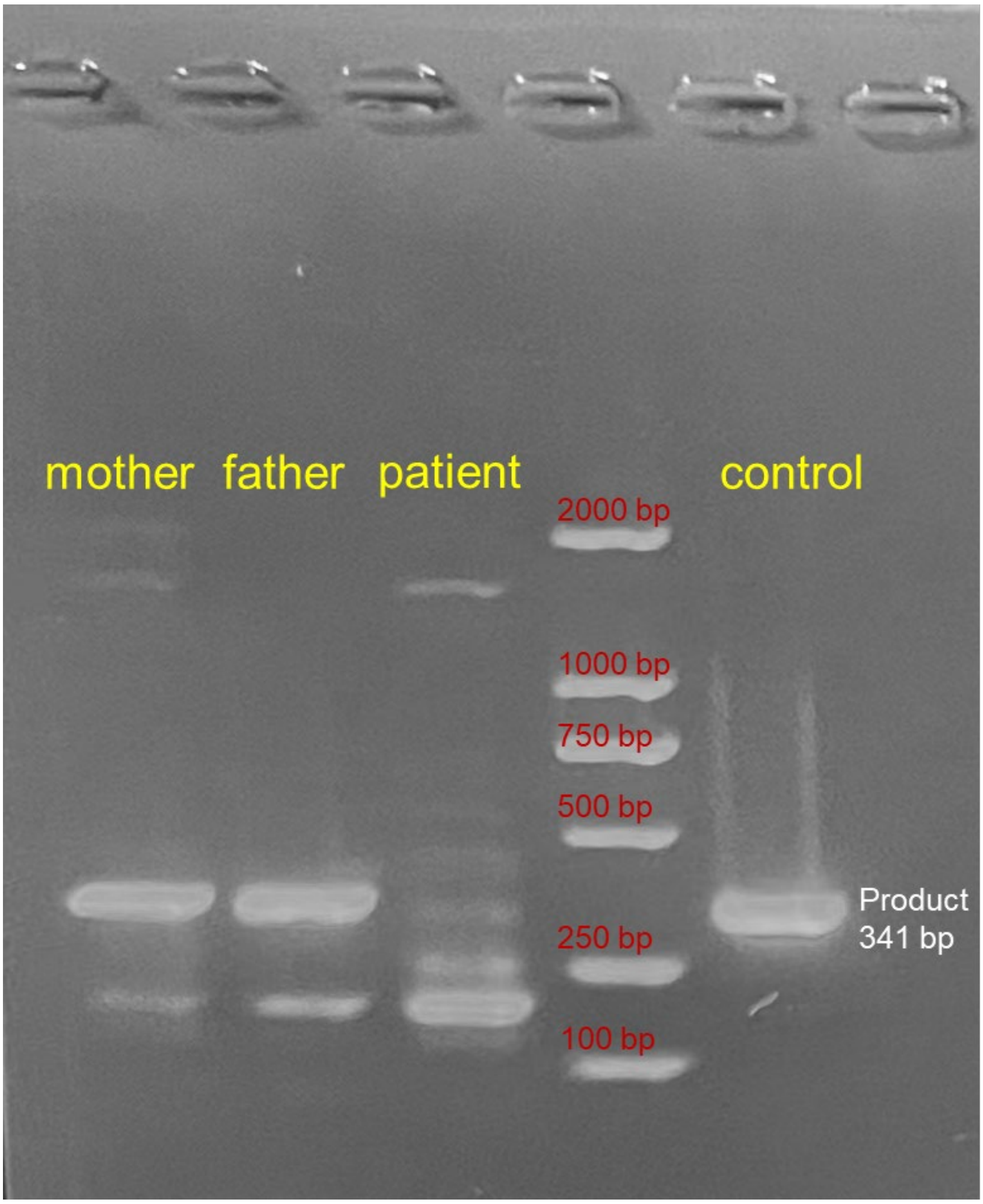

Supplementary Figure 2. The electrophoresis results for the exon2 showed the patient was homozygous (total depletion of *DRC1* exon2, only positive for the breakpoint PCR), while the patient's parents were heterozygous for the *DRC1* variant (both positive for the *DRC1* exon2 and the breakpoint PCR). The primer sequences designed for exon2 are listed as follows: forward primer: 5'-GATCTTGAACTCCTGGCCTTA-3', reverse primer: 5'-CAACATGGACCACATGATTTCT-3'.
